# Biochar, Compost, and Biochar–Compost Blend Applications Modulate Growth, Photosynthesis, Osmolytes, and Antioxidant System of Medicinal Plant *Alpinia zerumbet*

**DOI:** 10.3389/fpls.2021.707061

**Published:** 2021-08-23

**Authors:** Faisal Zulfiqar, Jianjun Chen, Adnan Younis, Zainul Abideen, Muhammad Naveed, Hans-Werner Koyro, Kadambot H. M. Siddique

**Affiliations:** ^1^Department of Horticultural Sciences, Faculty of Agriculture and Environment, The Islamia University of Bahawalpur, Bahawalpur, Pakistan; ^2^Environmental Horticulture Department and Mid-Florida Research and Education Center, Institute of Food and Agricultural Sciences, University of Florida, Apopka, FL, United States; ^3^Institute of Horticultural Sciences, University of Agriculture Faisalabad, Faisalabad, Pakistan; ^4^Dr. M. Ajmal Khan Institute of Sustainable Halophyte Utilization, University of Karachi, Karachi, Pakistan; ^5^Institute of Soil Sciences, University of Agriculture Faisalabad, Faisalabad, Pakistan; ^6^Institute of Plant Ecology, Justus-Liebig-University Giessen, Giessen, Germany; ^7^The UWA Institute of Agriculture, The University of Western Australia, Perth, WA, Australia

**Keywords:** antioxidant system, medicinal plant, organic amendments, Zingiberaceae, proline, glycine betaine

## Abstract

*Alpinia zerumbet* (Zingiberaceae) is a unique ornamental and medicinal plant primarily used in food ingredients and traditional medicine. While organic amendments such as biochar (BC) and compost (Co) have been demonstrated to improve plant productivity, no studies have examined their effects on the growth, physiology, and secondary metabolites of *A. zerumbet*. This study evaluated the impact of the amendment of BC, Co, or a biochar and compost mixture (BC+Co) on modifying and improving the growth, photosynthesis, antioxidant status, and secondary metabolism of *A. zerumbet* grown on sandy loam soil. The morpho-physiological and biochemical investigation revealed variation in the response of *A. zerumbet* to organic amendments. The amendment of BC and BC+Co significantly increased net photosynthetic rates of plants by more than 28%, chlorophyll *a* and *b* contents by 92 and 78%, respectively, and carboxylation efficiency by 50% compared with those grown in the sandy loam soil without amendment. Furthermore, the amendment significantly decreased plant oxidative stress, measured as leaf free proline and glycine betaine. Enzymatic antioxidant activity, total phenols, and flavonoids also varied in their response to the organic amendments. In conclusion, this study shows that BC and/or Co amendments are an efficient and sustainable method for improving the metabolite contents and reducing oxidative stress in *A. zerumbet*.

## Introduction

There is increasing interest in soil amendments, such as biochar (BC) and compost (Co) for the sustainable production of high-value medicinal plants and crops (Basak et al., 2021; Liu et al., 2021; Nigam et al., 2021). BC is a recalcitrant, black carbonaceous, porous, and low-density material produced by the pyrolysis of various biological residues, such as crop residue, wood waste, manure, food waste, forest residue, and sewage sludge (Wang et al., 2020). BC amendment to soils can improve the photosynthesis, growth, and yield of crop plants (Ali et al., 2019; He et al., 2020; Mansoor et al., 2020; Wang et al., 2021). Compost is an organic residue product produced by aerobic biological decomposition (biodegradation process). Compost amendment has been shown to alter plant metabolism, improving plant growth and productivity (Liang et al., 2021).

Plant metabolites, such as phenolics and flavonoids, are a vital group of active constituents of medicinal plants with therapeutic importance (Zulfiqar et al., 2021). These antioxidants play a crucial role in mitigating oxidative stress by regulating reactive oxygen species (Hasanuzzaman et al., 2020; Zulfiqar et al., 2021). The therapeutic properties of medicinal plants depend on these metabolites and antioxidant activity, which can be improved by amending soils with organic materials (Liu et al., 2021; Mehdizadeh et al., 2021; Nigam et al., 2021). BC application to medicinal plants has been reported to affect plant metabolites and the antioxidant system under stress conditions (Liu et al., 2021; Nigam et al., 2021). Similarly, soils amended with compost can influence these traits in medicinal plants (Burducea et al., 2019). The combined application of BC and Co could have additional benefits for improving medicinal plant production (Zulfiqar et al., 2019a). However, less is known about the interactive effects of the compost and BC application on the physiological and biochemical pathways of medicinal plants under non-stressed conditions.

This study considered shell ginger (*Alpinia zerumbet* (Pers.) B.L. Burtt. and R.M. Sm.), a member of the family Zingiberaceae, and a tall herbaceous crop that is widespread and important in tropical and subtropical regions of China, Taiwan, Japan, and Brazil. Shell ginger is commonly grown as a landscape and cut-foliage species. It has many pharmacological properties due to its chemical constituents, such as flavonoids, phenolic acids, phenylpropanoid glycosides, kava pyrones, sterols, and terpene (Lim, 2016; Chan et al., 2017). For instance, the fruits are used to treat gastrointestinal and cardiovascular diseases, rhizomes are consumed as spices, stem fibers are used to produce paper, kariyushi wear, and textiles, and the essential oil from leaves is used in cosmetics (e.g., perfume, soap, skincare, and deodorant) and insect repellent. To determine the beneficial effects of BC and Co on shell ginger, we investigated the effect of BC and Co, individually and combined, on the growth, physiology, and secondary metabolites of shell ginger, which will be of interest for both growers and the pharmaceutical industry.

## Materials and Methods

### Experimental Location and Plant Material

The experiment was established in the floriculture research area of the Institute of Horticulture, University of Agriculture Faisalabad, Pakistan (31°300 N, 73°100 E, altitude 213 m), under natural daylight conditions in typical subtropical climate conditions from April 12, 2017 to March 20, 2018. A 40% shading net was placed above the metal canopy to prevent high light intensities and temperatures on sunny days. *Alpinia* plants (with 18-cm height, 85-g fresh weight, six-leaf stage) produced from tissue culture were purchased from a local nursery in Pakistan (Best Garden Nursery, Faisalabad).

### Selection and Preparation of Raw Material

Sandy loam soil (65 sand, 20 silt, and 15% clay) was taken from the top layer (~6–22 cm depth) of a field (31°300 N, 73°100 E, altitude 213 m), air-dried, ground, and sieved.

Biochar was produced by the slow pyrolysis of chopped wheat straw (*Triticum aestivum* L.) prepared at 450°C for 2-h resident time. Prior to the soil amendment, the BC was air-dried for 4 days and grounded. Compost was manufactured from plant leaf residue (the Institute of Horticultural Sciences, University of Agriculture Faisalabad, Pakistan) and sieved to <2mm. The compost and/or BC were mixed with the soil before pot filling. Plants were grown in earthen pots (with 28-cm top diameter, 24-cm base diameter, and 22-cm height). The soil, BC, and Co properties are listed in Table 1.

**Table 1:** Chemical characteristics and nutritional composition of wheat straw biochar, compost, and soil used in the study.

**Chemical properties**	**Soil**	**Biochar**	**Compost**
pH	7.56	7.94	6.89
Electrical conductivity (dS m^−1^)	1.15	2.75	1.2
Cation exchange capacity (cmol_c_ kg^−1^)	5.69	82.40	90
Moisture (%)	23	4.21	ND
Organic matter (%)	0.70	–	–
Carbon (g kg^−1^)	–	633.51	199.2
Nitrogen (g kg^−1^)	–	11.25	21.8
Total phosphorus (g kg^−1^)	–	1.43	0.42
Total potassium (g kg^−1^)	–	9.29	1.67
Zinc (mg kg^−1^)	–	47.61	47.0
Iron (mg kg^−1^)	–	85.31	70.8

### Preparation of Soil Potting Mixtures

Four different soil mixtures were tested as follows: (1) soil only as control (S), (2) BC soil amendment (5% by weight), (3) Co soil amendment (5% by weight), and (4) BC+Co (5 + 5% by weight).

### Experimental Setup

The experiment had a randomized block design with four replications. The median light intensity received by *A. zerumbet* plants was about 150 μmol photons m^−2^ s^−1^, with a 95% quantile of 1,230 μmol photons m^−2^ s^−1^. The median temperature was about 25°C. Plants were tap-watered by hand on alternate days. Seedlings were provided with a half-strength Hoagland nutrient medium after 7 days. The daily photoperiod ranged from 14 to 16 h. The impact of the soil amendments on growth, gas exchange, photosynthetic pigments, and antioxidants was studied for 12 months.

### Plant Sampling and Analysis

Morphological characteristics, gas exchanges, chlorophyll contents, some secondary metabolites, and activities of the oxidative-related enzymes of shell ginger plants grown in the four treatments were examined.

### Plant Growth Parameters

The non-destructive vegetative parameters, such as plant height, tiller number, shoot diameter, and leaf number, were recorded at the end of the experiment (March 2018). Plant height was determined as the distance from the root base to the top of the plant. The main shoot diameter was measured with a digital Vernier caliper (India Tools and Instruments Co., Mumbai, India). Four plants (*n* = 4) in each treatment were separated into leaf and root tissue, and the fresh mass of each was measured with a digital balance. Leaf area was determined with an LI-3000C portable area meter (LI-COR, Lincoln, NE, USA) and subsequently used to calculate the leaf area/leaf mass ratio [specific leaf area (SLA)].

### Gas Exchange Measurements

Gas exchange parameters were measured on March 12, 2018. Photosynthetic rate (*P*_*n*_), stomatal conductance (*g*_*s*_), transpiration rate (*E*), internal CO_2_ concentration (*C*_*i*_), water use efficiency (*P*_*n*_**/***E*), and carboxylation efficiency (COE, *P*_*n*_**/**CO_2_) concentration were analyzed on fully expanded mature leaves (i.e., midportion; two per replicate) on a sunny day between 10:00 and 12:30 with a portable CO_2_ IR gas analyzer (Analytical Development Company, Hoddesdon, England).

### Chlorophyll Contents

Eight days before the end of the experiment (March 12, 2018), two mature leaves per plant were excised from the middle portion of the main shoot (i.e., six replicates per treatment); 0.5 g was mortared and kept overnight (dark) in 80% acetone at −4°C. The extract was centrifuged (Z 306; HERMLE Labortechnik, Wehingen, Germany) at 10,000 × *g* for 5 min. The absorbance of the supernatant was read at 663 and 645 nm using a UV-1900 spectrophotometer (BMC, Canada). Chlorophyll concentrations (i.e., *a, b*, and total chlorophyll) were calculated following the protocol of Arnon (1949).

### Leaf Free Proline Content

A mature leaf sample (0.5 g) was isolated from the middle portion of a plant (i.e., four replicates per treatment) and mixed with 10 ml of 3% (w/v) sulfosalicylic acid (MP Biomedicals, Inc., Solon, OH, USA) according to the study by Bates et al. (1973). The samples were filtered; 2.0 ml filtrate was mixed with 2.0 ml acid ninhydrin solution; and 2.0 ml glacial acetic acid (GAA) (MP Biomedicals, Inc.) in a test tube. Acid ninhydrin was prepared by mixing 1.25 g ninhydrin (C6H4COCOCOH2O, BDH, Anala R, England) with 30 ml GAA and 20 ml of 6 M H_3_PO_4_. The optical density of the filtrate was measured at 520 nm using a UV-1900 spectrophotometer (BMC, Canada).

### Glycine Betaine Content

Leaf material (0.5 g) for each replicate was shaken occasionally in 10 ml toluene (0.5%) and kept at 4°C overnight. After filtration and centrifugation, 1 ml filtrate was added to 1 ml of 2 N sulfuric acid and 200 μl potassium triiodide (KI_3_) in a test tube. Samples were cooled at 4°C for 1 h in a chiller before adding 2.8 ml ice-cooled deionized H_2_O and 5 ml 1,2-dichloroethane. The absorbance of the organic layer (lower layer) was recorded spectrophotometrically (UV-1900 spectrophotometer; BMC, Canada) at 365 nm. The glycine betaine (GB) concentrations were recorded against a standard curve following the study by Grieve and Grattan (1983).

### Preparation of Extracts for Biochemical Assays

Fresh leaf material (1 g) was pulverized in a mortar and extracted with 25 ml solvent (i.e., methanol, ethanol, acetone, or water). An orbital shaker (Kalsterin, YR 40, Montpellier, France) was used to continually stir at 150 × *g* for 24 h to homogenize the extract before centrifuging at 10,000×*g* for 10 min. The supernatant was filtered using the Whatman filter paper (No. 1) (Tisch Scientific, Cleves, OH, USA). The filtrate was used for further screening.

### Quantification of Total Phenolic Content

The total phenolic content (TPC) of the leaf extract was quantified at 765 nm using a UV-1900 spectrophotometer (BMC, Canada), as described by Folin and Ciocalteu (1927). Briefly, 1 ml leaf extract was mixed with 4 ml sodium carbonate (20%) and 5 ml Folin–Ciocalteu solution (10%) and incubated for 1 h in a water bath. Absorbance was read at 765 nm (Optizen POP, Mecasys Co., Ltd., Korea). The TPC was expressed as gallic acid equivalents (GAE). Of note, 1 ml of each standard was mixed with 4 ml sodium carbonate (20%) and 5 ml Folin–Ciocalteu reagent, and the absorption was measured after 1 h at 765 nm using a UV-1900 spectrophotometer (BMC, Canada). A calibration curve was prepared using absorbance as a function of concentration. Finally, GAE was calculated as follows:

$$\begin{array}{l} \text{T}=\text{C}\times \text{V}/\text{M} \end{array}$$

where T is the TPC (mg GAE per g plant extract), C is the gallic acid concentration determined from the calibration curve (mg ml^−1^), V is the volume of extract (ml), and M is the weight (g) of pure plant extract.

### Determination of Total Flavonoid Content

The total flavonoid content (TFC) of the prepared leaf extract was estimated using the AlCl_3_ colorimetric method described by Chang et al. (2006) using quercetin as a standard. The extract or standard solution (0.5 ml) was mixed with 2 ml distilled water and 0.15 ml of 5% NaNO_2_ solution. After 6 min of incubation, 0.15 ml of 10% AlCl_3_ solution was added. After 6 min, 1 M NaOH was added to the mixture. Finally, 3 ml methanol was added for an end volume of 5 ml. The reaction mixture was mixed thoroughly and incubated at room temperature for 45 min. Absorbance was measured using a UV-1900 spectrophotometer (BMC, Canada) at 510 nm. The TFC was expressed as catechin equivalents from the linear regression curve of catechin.

### Antioxidant Assays

#### Determination of 2,2-Diphenyl-1-Picrylhydrazyl (DPPH) Radical Scavenging Activity

Total free radical scavenging activity was measured using 2,2-diphenyl-1-picrylhydrazyl (DPPH) as per the method described by Yen and Chen (1995). Briefly, 0.5 g leaf tissue was homogenized in 10 ml acetone. Then, 3 ml leaf extract (i.e., three replicates) was added to 1 ml DPPH methanol solution (0.004%), vigorously shaken, and incubated in the dark for 30 min at room temperature. Absorbance was read at 517 nm using a UV-1900 spectrophotometer (BMC, Canada). A low absorbance reading indicates high radical scavenging activity. DPPH inhibition was calculated as follows:

$$\begin{array}{l} \text{DPPH inhibition }\left(\%\right)\text{=}[(\text{Absorbance of blank- Absorbance}\\ \text{ of sample/Absorbance of blank})\times 100] \end{array}$$

#### Determination of Reducing Power Assay

The reducing power capacity of the leaf extract was evaluated by direct electron donation to reduce Fe^3+^ (CN)_6_ to Fe^2+^ (CN)_6_ using the method described by Yadav et al. (2014). Briefly, 1 ml prepared extract was mixed with 2.5 ml potassium ferricyanide (1%) and 2.5 ml phosphate buffer (0.2 M, pH 6.6). The mixture was incubated in a water bath at 50°C for 20 min before adding 2.5 ml trichloroacetic acid (1% w/v). The reaction mixture was centrifuged at 3,000 × *g* for 10 min. The supernatant (2.5 ml) was mixed with 0.5 ml ferric chloride (0.1%) and 2.5 ml deionized water. Absorbance was read at 700 nm using a UV-1900 spectrophotometer (BMC, Canada).

### Enzymatic Antioxidant Extraction and Assay

Leaf sample (0.5 g) was homogenized in 4 ml sodium phosphate buffer (0.05 M, pH 7.8), comprising 2% (w/v) polyvinylpyrrolidone (PVP) and 1.0 mM ethylenediaminetetraacetic acid (EDTA). The homogenate was centrifuged (Z 306; HERMLE Labortechnik, Wehingen, Germany) at 10,000 × *g* for 17 min at 4°C. The supernatant (i.e., protein extract) was used in the catalase (CAT) and peroxidase (POD) assays and to determine total soluble proteins (TSP).

### Catalase Activity

Catalase activity was determined according to the method described by Chance and Maehly (1955). Briefly, 0.1 ml of the reaction mixture contains 0.9 ml of 5.9 mM H_2_O_2_, 2 ml of 50 mM phosphate buffer, and 0.1 ml protein extract. The changes in absorbance were measured at 240 nm using a UV-1900 spectrophotometer (BMC, Canada). Absorbance was read at 30-s intervals for 5 min to study H_2_O_2_ decomposition, reflecting CAT activity (μmol min^−1^ mg^−1^ protein).

### Peroxidase Activity

Peroxidase activity was determined in leaf extracts using the method of Chance and Maehly (1955). The reaction mixture contained 0.1 ml protein extract, 0.4 ml guaiacol (20 mM), 0.5 ml H_2_O_2_ (40 mM), and 2 ml sodium phosphate buffer (50 mM). Absorbance was read at 470 nm in 20-s intervals with a UV-1900 spectrophotometer (BMC, Canada). The absorbance slope was used to calculate POD activity (μmol min^−1^ mg^−1^ protein).

### Superoxide Dismutase Activity

Superoxide dismutase activity was assayed using the method of Van Rossum et al. (1997). The reaction mixture contained 0.4 ml distilled water, 0.1 ml methionine, 0.1 ml Triton-X, 0.25 ml phosphate buffer (pH 7.8), 0.5 ml nitro blue tetrazolium, 0.5 ml riboflavin, and 0.5 ml protein extract. The mixture was kept in the light for 20 min before recording the absorbance at 560 nm.

### Total Soluble Proteins

Total soluble proteins were determined according to the protocol described by Bradford (1976). The absorbance of the prepared reaction mixture (0.2 ml protein extract, 0.02 ml Coomassie blue dye, and 0.78 ml deionized water) was read at 595 nm with a UV-1900 spectrophotometer (BMS, Canada).

### Statistical Analysis

All collected data were subjected to ANOVA using SPSS software version 11.0 (SPSS, Chicago, IL, USA). If significance occurred among treatments, means were separated by the least significance difference (LSD) at *p* < 0.05 level. SigmaPlot software version 12.0 (Systat, San Jose, CA, USA) was used to display the means and SE of the dataset.

## Results

### Effect of Organic Amendments on Plant Growth

**Figure 1:**
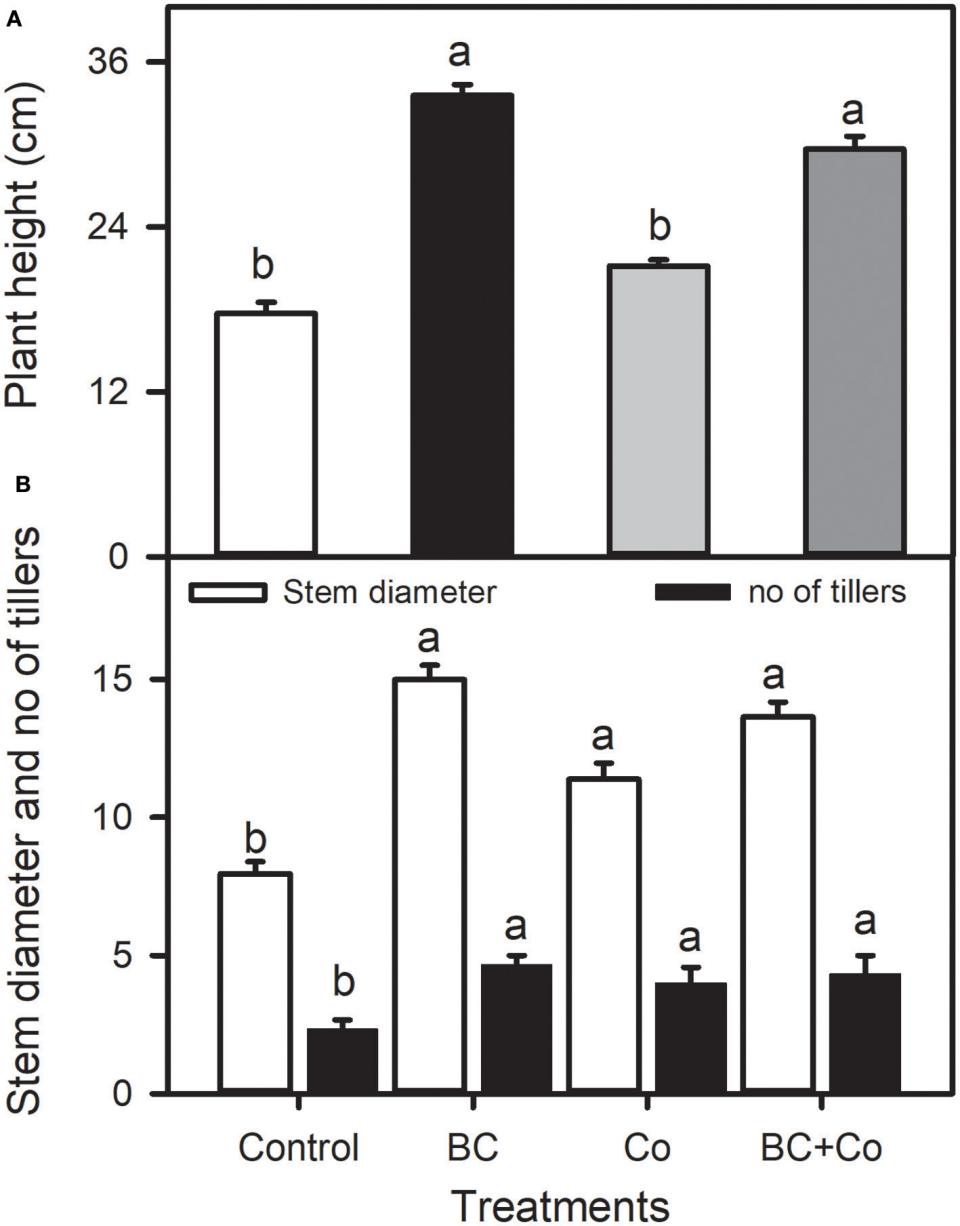
Plant height **(A)**, and stem diameter and tiller number **(B)** of *Alpinia zerumbet* treated with biochar (BC), compost (Co), and biochar+compost mixture (BC+Co). Data are the mean ± SE (*n* = 6). Different letters above the bars represent significant differences according to the least significance difference (LSD) *post hoc* test at the *p* < 0.05 level.

The application of BC and/or Co significantly affected the measured growth parameters of plants. The BC and BC+Co treatments increased plant height by 40 and 47%, respectively, relative to the control (Figure 1). The BC, Co, and BC+Co treatments increased tiller number (about two-fold) and stem diameter, compared to the control, more so in the two BC treatments (Figure 1). Leaf fresh weights increased in the three amended treatments, relative to the control, more so in the BC+Co treatment (Table 2). The BC and BC+Co treatments increased leaf numbers by 65% compared to the control. Leaf areas of plants grown in the three amendment media were more than doubled with respect to those of plants grown in the control medium. The SLA of plants grown in BC+Co was the highest among the treatments (Table 2).

**Table 2:** Impact of organic amendments (biochar, compost, and biochar+compost) on growth parameters (means ± SE) of *Alpinia zerumbet*.

**Treatments**	**Control**	**BC**	**Co**	**BC+Co**
Leaf fresh weight (g)	7.25 ± 0.1c	9.57 ± 0.2b	12.65 ± 0.6b	15.45 ± 0.0a
Leaf number	8.66 ± 0.2c	18.66 ± 0.3a	11.66 ± 0.3b	14.33 ± 0.1b
Leaf area (cm^2^)	28.43 ± 0.5c	55.97 ± 1.9a	40.33 ± 0.5b	46.23 ± 1.1b
Specific leaf area (cm^2^/g)	3.92 ± 0.1c	8.53 ± 0.4b	11.02 ± 0.1a	13.39 ± 0.2a
Root fresh mass (g)	109 ± 2.0b	100 ± 1.8b	111± 4.3b	137 ± 2.6a

*BC, Biochar; Co, Compost. Means followed by the same letter in each row do not significantly differ (p ≤ 0.05)*.

### Effect of Organic Amendments on Leaf Gas Exchange

Net photosynthesis rate (*P*_*n*_) increased by 19 and 26% in the BC and BC+Co treatments, respectively, compared to control (Table 3). The Co treatment did not significantly increase *P*_*n*_, relative to the control (Table 3). Stomatal conductance (*g*_*s*_) decreased (32%) sharply only in the Co treatment. Internal CO_2_ concentration declined in the three amended treatments, relative to the control. COE increased by 15% in the BC and BC+Co treatments. Transpiration rate (*E*) increased by 40, 12, and 57%, respectively, in the BC, Co, and BC+Co treatments, relative to the control (Table 3).

**Table 3:** Impact of organic amendments (biochar, compost, and biochar+compost) on leaf gas exchange and chlorophyll contents (means ± SE) of *A. zerumbet*.

**Treatments**	**Control**	**BC**	**Co**	**BC+Co**
Net photosynthesis (*P_*n*_*)	5.58 ± 0.41b	7.16 ± 0.68a	5.96 ± 0.60b	7.74 ± 1.32a
Stomatal conductance (*g_*s*_*)	0.63 ± 0.08a	0.56 ± 0.08b	0.43 ± 0.12c	0.60 ± 0.05a
Internal carbon dioxide (*C_*i*_*)	263 ± 3.52a	229 ± 3.52b	222 ± 1.73b	218 ± 2.33b
Transpiration (*E*)	0.49 ± 0.16d	1.00 ± 0.30b	0.68 ± 0.11c	1.40 ± 0.25a
Carboxylation efficiency (*P_*n*_*/*C_*i*_*)	0.002 ± 0.00c	0.031 ± 0.00a	0.02 ± 0.01b	0.03 ± 0.01a
Chlorophyll *a*	1.15 ± 0.08c	2.21 ± 0.10a	1.76 ± 0.13b	2.55 ± 0.29a
Chlorophyll *b*	0.64 ± 0.03c	1.14 ± 0.08b	0.87 ± 0.04c	1.46 ± 0.02a
Total chlorophyll	1.80 ± 0.10c	3.35 ± 0.09a	2.63 ± 0.10b	4.01 ± 0.32a

*BC, Biochar; Co, Compost; P_n_, μmol CO_2_ m^−2^ s^−1^; g_s_, mol H_2_O m^−2^ s^−1^; C_i_, μmol m^−2^ s^−1^; E, mmol H_2_O m^−2^ s^−1^; P_n_/C_i_, mol m^−2^ s^−1^; Chlorophyll a, b, and total, mg g^−1^ FW. Means followed by the same letter in each row do not significantly differ (p ≤ 0.05)*.

### Effect of Organic Amendments on Chlorophyll Contents

Chlorophyll *a, b*, and total chlorophyll contents increased in the three amended treatments, more so in the BC+Co treatment, relative to the control (Table 3).

### Effect of Organic Amendments on Biochemical Parameters

**Figure 2:**
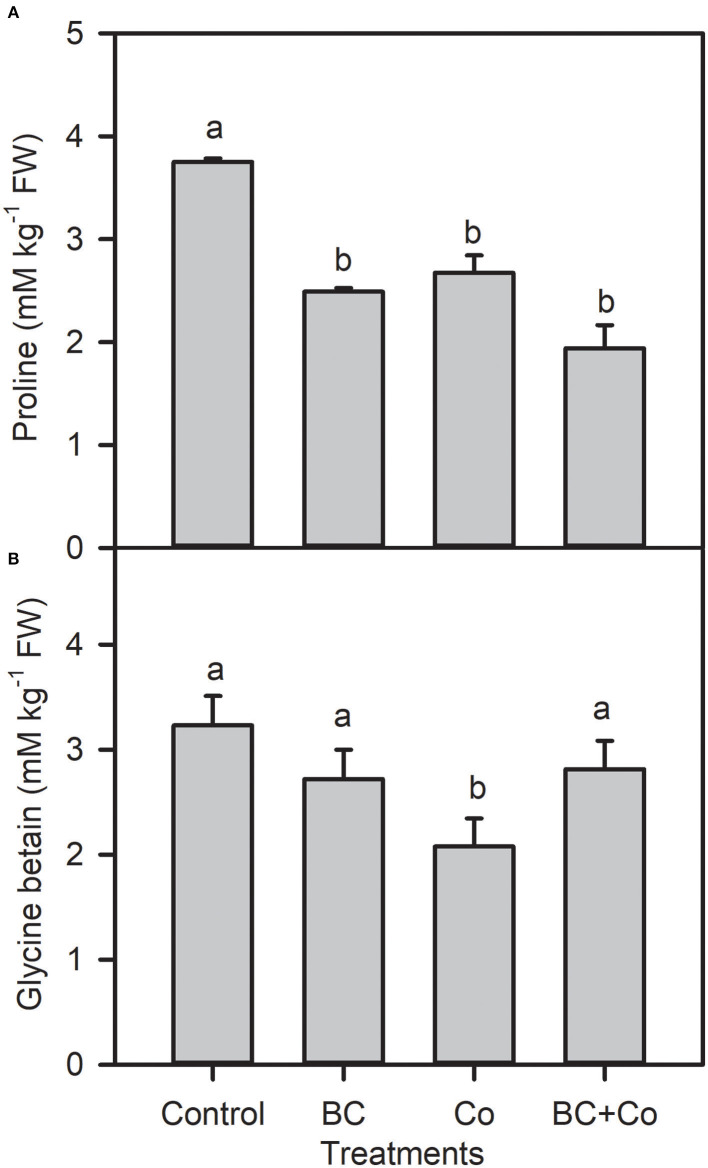
Leaf proline **(A)** and glycine betaine **(B)** contents of *A. zerumbet* treated with BC, Co, and BC+Co. Data are the mean ± SE (*n* = 6). Different letters above the bars represent significant differences according to the LSD *post hoc* test at the *p* < 0.05 level.

**Figure 3:**
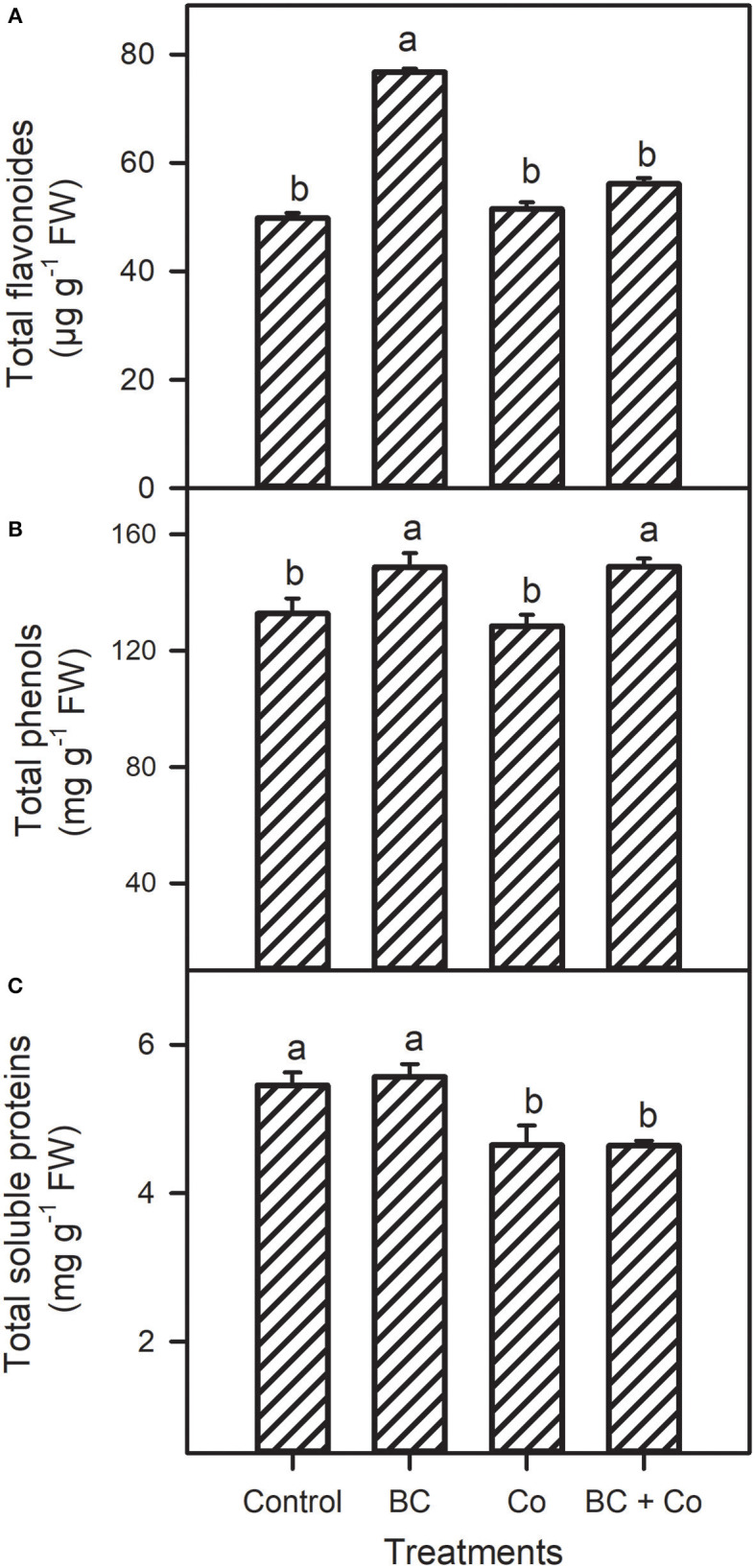
Leaf total flavonoids **(A)**, phenolics **(B)**, and soluble proteins **(C)** of *A. zerumbet* treated with BC, Co, and BC+Co. Data are the mean ± SE (*n* = 6). Different letters above the bars represent significant differences according to the LSD *post hoc* test at the *p* < 0.05 level.

Leaf free proline and GB contents showed a differential decrease response in the three amended treatments, relative to the control (Figure 2). The TFC increased 1.5-fold in the BC treatment but did not change in the Co or BC+Co treatments, compared to the control (Figure 3). TPC increased by 15% in the BC and BC+Co treatments, but the Co treatment did not influence this trait, relative to the control. The TSP in plants grown in BC were similar to those grown in the control medium but decreased in the Co and BC+Co treatments by 15%, relative to the control (Figure 3).

**Figure 4:**
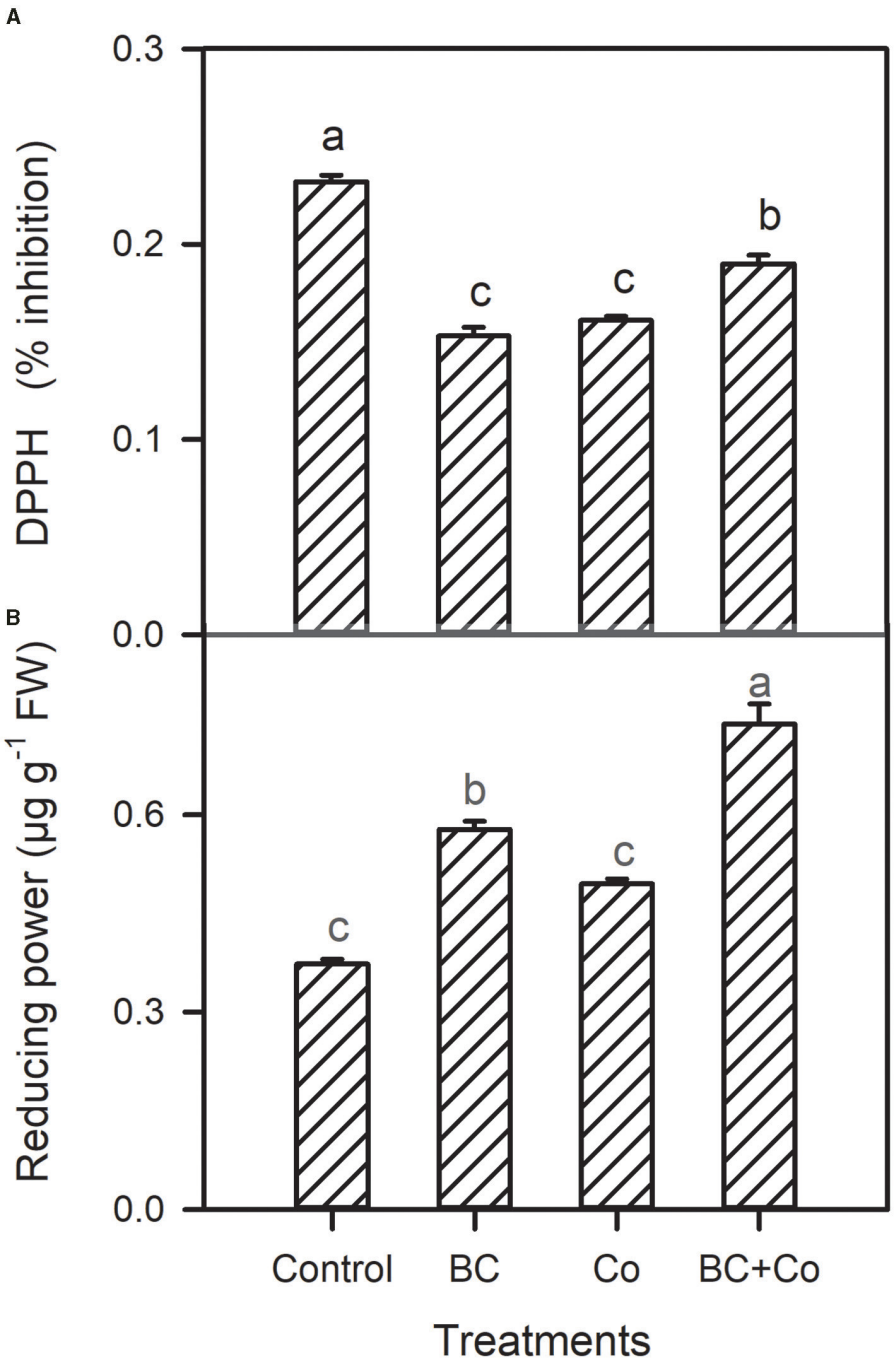
Leaf DPPH % inhibition **(A)** and reducing power **(B)** of *A. zerumbet* plants treated with BC, Co, and BC+Co. Data are the mean ± SE (*n* = 6). Different letters above the bars represent significant differences according to the LSD *post hoc* test at the *p* < 0.05 level.

**Figure 5:**
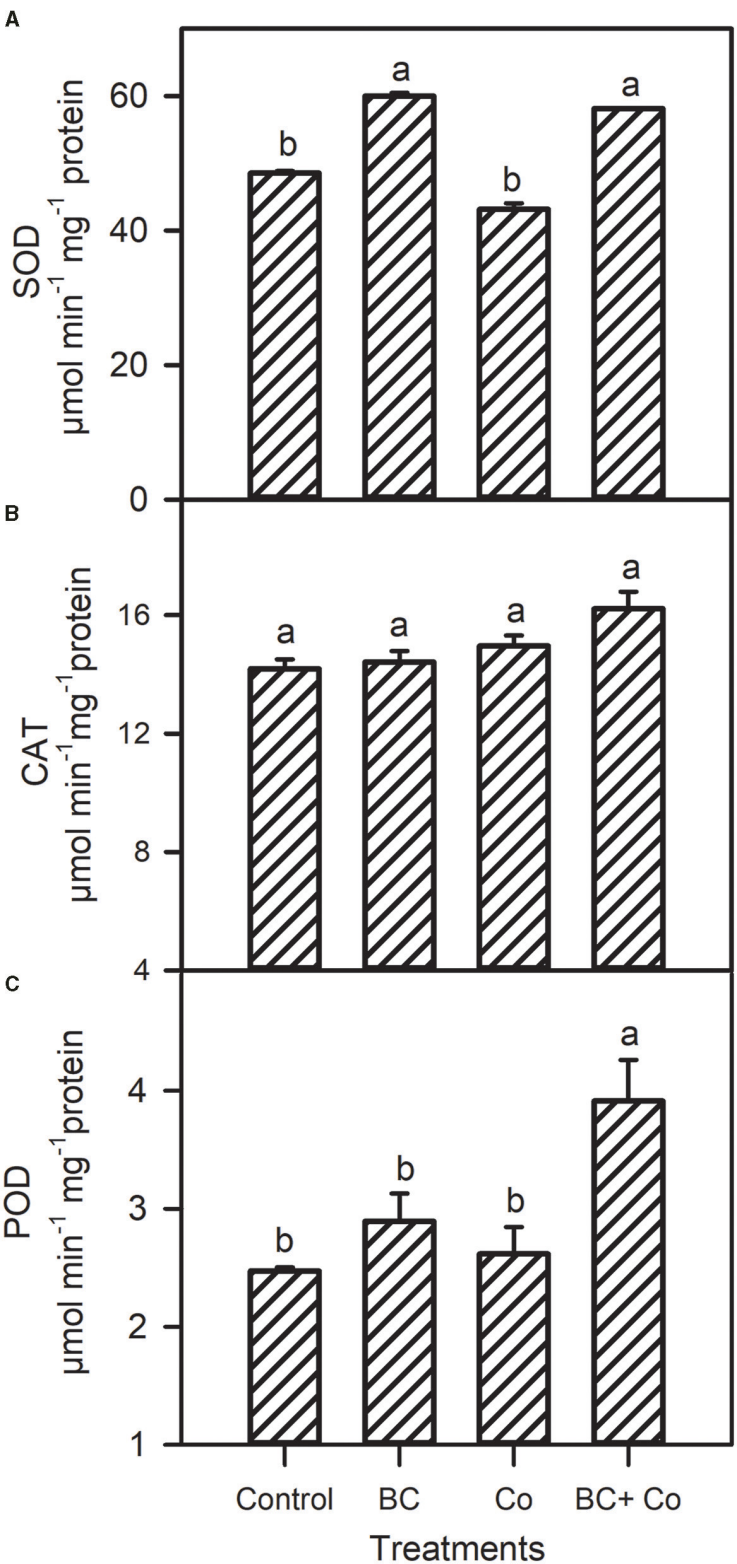
Superoxide dismutase (SOD) **(A)**, catalase (CAT) **(B)**, and peroxidase (POD) **(C)** antioxidant enzyme activities of *A. zerumbet* plants treated with BC, Co, and BC+Co. Data are the mean ± SE (*n* = 6). Different letters above the bars represent significant differences according to the LSD *post hoc* test at the *p* < 0.05 level.

Antioxidant capacity by DPPH declined in the three amended treatments, relative to the control. The antioxidant activity by reducing power assay increased in the BC and BC+Co treatments more than the Co treatment (Figure 4). The CAT activity did not change in any treatment, with respect to the control, while POD increased by 60% and SOD activities increased by 18% in plants grown in BC+Co medium compared with those grown in the control medium. SOD of plants grown in BC also increased by 18% compared to the control medium (Figure 5).

## Discussion

### Plant Growth

The application of BC, Co, and BC+Co increased total biomass in *A. zerumbet*. The increase in plant height, tiller number, stem diameter, and leaf and root fresh weights in response to organic amendments could be associated with enhanced photosynthesis, as reported elsewhere (Agegnehu et al., 2016; Singh et al., 2018; Sánchez-Monedero et al., 2019; Rasool et al., 2021a). In contrast, Schmidt et al. (2014) reported a small and mostly nonsignificant effect of BC and combined applications of BC and Co on grapevines grown in nutrient-poor soil.

The organic soil amendment increased leaf fresh weight in *A. zerumbet* due to changes in various leaf-related parameters, such as an increased leaf canopy and foliage area to facilitate net photosynthesis, particularly in the BC+Co treatment. In contrast, the presence of BC and Co in the growth medium had a smaller increase in leaf fresh weight.

The BC+Co treatment improved root development in *A. zerumbet* more than BC or Co alone. This is consistent with previous studies where combined BC and Co application increased soil moisture and nutrient retention and thus root growth more than individual applications (Sorrenti et al., 2019; Abideen et al., 2020a; Teodoro et al., 2020). The positive effect of BC on root growth could be due to changes in physical soil condition, such as soil pH, water holding capacity, and hormonal effects, thus accelerating root growth and improving overall growth (Somerville et al., 2020).

### Physiological Attributes

Higher photosynthetic rates in *A. zerumbet* were observed in the BC (19%) and BC+Co (26%) treatments than the compost treatment, which was similar to the control and could be related to improved COE at decreased intracellular CO_2_ concentration. Other studies have also reported higher *P*_*n*_ with BC and BC+Co amendments relative to the control (Xu et al., 2015; Seehausen et al., 2017).

The BC+Co mixture increased transpiration the most in *A. zerumbet*, while Co alone had no effect. BC alone and combined increased soil water availability and soil water holding capacity, thus increasing transpiration. Hence, higher transpiration was associated with higher *P*_*n*_ for *Alpinia* plants grown in BC+Co. Plants grown in soil amended with BC and Co can minimize substantial water losses through stomatal closure and transpiration (Kammann and Graber, 2015), which helps maintain water balance and leaf turgidity. Thus, organic soil improvements ultimately support photosynthetic performance (Xu et al., 2015), while decreasing *C*_*i*_, *C*_*i*_/*C*_*a*_, and *g*_*s*_.

Chlorophyll *a* and *b* contents of *Alpinia* increased in the BC and BC+Co treatments, as reported for *Phragmites karka* (Agegnehu et al., 2016). Higher chlorophyll contents are accompanied by higher *P*_*n*_ in plants grown in BC and BC+Co. The high chlorophyll contents in *Alpinia* could be related to improved soil physical and chemical properties that facilitated the plant absorption of nutrients, especially nitrogen concentration (Almaroai and Eissa, 2020), and may increase light absorption to increase photosynthesis. Increased chlorophyll contents act as a proxy for leaf maximum carboxylation rate, which could be supported by increased water flux and carbon acquisition with BC and Co application to soil. Increased chlorophyll in the presence of BC and Co triggers nitrogen supply for photosynthetic enzymes and indirectly enhances COE (Luo et al., 2018).

### Biochemical Parameters

Total flavonoid and polyphenol contents increased in the BC treatments but were unaffected or decreased in the sole Co treatment. The synthesis of phenolic compounds was associated with higher antioxidant activity (reducing power activity), which helps plants detoxify reactive oxygen species, indicating that BC is useful for enhancing plant antioxidant capability and protecting plants from oxidative stress by increasing phenolic acid concentrations. The antioxidative balance is important for optimal leaf photosynthesis and biomass production, as reflected in the higher POD and SOD activities in *Alpinia* amended with BC+Co, which increased plant biomass. Plant antioxidants act as a natural defense system against various stresses that induce excessive production of reactive oxygen species (Ahmad et al., 2010, 2019; Kohli et al., 2019; Zulfiqar and Ashraf, 2020). Numerous studies have shown that organic amendments improve the systematic resistance of antioxidant enzymes in plants (Quartacci et al., 2017; Rehman et al., 2019; Zulfiqar et al., 2019b; Abideen et al., 2020a,b; Rasool et al., 2021b).

Plants subjected to climatic variations could accumulate osmotic substances in cells, such as proline, soluble sugars, and various betaines, which function as osmoprotectants (Zulfiqar et al., 2020). In *Alpinia*, the soil organic amendments decreased proline and GB accumulation due to osmoprotectant mechanisms. The accumulation of nitrogenous compounds also declined, which was related to strong enzymatic and non-enzymatic defense in *Alpinia*. An elevated plant antioxidative defense system is related to reduced energy demands for catabolism and increased demand for anabolism (biomass synthesis) with BC and Co amendment (Ali et al., 2017; Quartacci et al., 2017; Huang et al., 2019).

## Conclusions

Organic soil amendments (i.e., BC, Co, and BC+Co) had a positive effect on the growth and physiobiochemical response of *A. zerumbet*. In particular, BC+Co significantly increased the growth, chlorophyll content, photosynthesis, and antioxidant defense system activity, and it also reduced the proline and GB accumulation (Table 4). The results support the view that BC alone or combined with Co alters the physiobiochemical characteristics of *A. zerumbet*. Further research studies are needed to evaluate the agronomic and environmental benefits of *A. zerumbet* supplied with different feedstock-based BCs at different concentrations and combined with compost, especially under field conditions.

**Table 4:** Representation of changes in different parameters of *A. zerumbet* after treatment with biochar, compost, and biochar+compost.

**Parameters**	**Treatments**		
	**BC**	**Co**	**BC+Co**
Growth	↑	↑	↑↑
Net photosynthesis	↑	↓	↑
Leaf free proline	↓	↓	↓
Glycine betaine	↓	↓↓	↓
Total phenolic contents	↑	–	↑
Total flavonoid content	↑	–	–
Total soluble proteins	–	↓	↓
DPPH radical scavenging activity	↓↓	↓↓	↓
Reducing power assay	↑	↑	↑↑
Superoxide dismutase	↑	↓	↑
Peroxidase activity	↑	–	↑
Catalase activity	–	–	↑

*BC, Biochar; Co, Compost. The direction of the arrow shows the change relative to the control treatment. The number of arrows shows the intensity of response*.

## Data Availability Statement

The raw data supporting the conclusions of this article will be made available by the authors, without undue reservation.

## Author Contributions

FZ designed and undertook the experiment, contributed to the collection and interpretation of data, and drafted the manuscript. All authors contributed to editing and revising the manuscript, and they read and approved the final manuscript.

## Conflict of Interest

The authors declare that the research was conducted in the absence of any commercial or financial relationships that could be construed as a potential conflict of interest.

## Publisher's Note

All claims expressed in this article are solely those of the authors and do not necessarily represent those of their affiliated organizations, or those of the publisher, the editors and the reviewers. Any product that may be evaluated in this article, or claim that may be made by its manufacturer, is not guaranteed or endorsed by the publisher.
